# Detecting Triple-Vessel Disease with Cadmium Zinc Telluride-Based Single-Photon Emission Computed Tomography Using the Intensity Signal-to-Noise Ratio between Rest and Stress Studies

**DOI:** 10.1155/2017/4945680

**Published:** 2017-10-15

**Authors:** Yu-Hua Dean Fang, Tzu-Pei Su, Chi-Jen Chang, Kung-Chu Ho, May Su, Tzu-Chen Yen

**Affiliations:** ^1^Department of Biomedical Engineering, National Cheng Kung University, Tainan, Taiwan; ^2^Department of Nuclear Medicine, Chang Gung Memorial Hospital, Keelung, Taiwan; ^3^Department of Cardiology, Chang Gung Memorial Hospital, Linkou, Taiwan; ^4^Department of Nuclear Medicine, Chang Gung Memorial Hospital, Linkou, Taiwan; ^5^Center for Advanced Molecular Imaging and Translation & Cyclotron Center, Chang Gung Memorial Hospital, Linkou, Taiwan

## Abstract

The purpose of this study was to investigate if a novel parameter, the stress-to-rest ratio of the signal-to-noise ratio (RSNR) obtained with a cadmium zinc telluride (CZT) SPECT scanner, could be used to distinguish triple-vessel disease (TVD) patients.* Methods*. One hundred and two patients with suspected coronary artery disease were retrospectively involved. Each subject underwent a Tl-201 SPECT scan and subsequent coronary angiography. Subjects were separated into TVD (*n* = 41) and control (*n* = 61) groups based on coronary angiography results using 50% as the stenosis cutoff. The RSNR was calculated by dividing the stress signal-to-noise ratio (SNR) by the rest SNR. Summed scores were calculated using quantitative perfusion SPECT (QPS) for all subjects.* Results*. The RSNR in the TVD group was found to be significantly lower than that in the control group (0.83 ± 0.15 and 1.06 ± 0.17, resp.; *P* < 0.01). Receiver-operating characteristic (ROC) analysis showed that RSNR can detect TVD more accurately than the summed difference score with higher sensitivity (85% versus 68%), higher specificity (90% versus 72%), and higher accuracy (88% versus 71%).* Conclusion*. The RSNR may serve as a useful index to assist the diagnosis of TVD when a fully automatic quantification method is used in CZT-based SPECT studies.

## 1. Introduction

Single-photon emission computed tomography (SPECT) myocardial perfusion imaging (MPI) is the most common noninvasive imaging modality used to evaluate myocardial perfusion, with approximately 5 million examinations performed annually in the USA alone [1]. Using perfusion tracers such as Tl-201 and ^99m^Tc-sestamibi, SPECT MPI is easy to perform at reasonable cost and with satisfactory image quality. However, SPECT MPI has limited ability to detect triple-vessel disease (TVD) [2, 3]. TVD is a high-risk condition found in 5%–10% of patients of coronary artery disease and often requires immediate intervention with stenting or bypass surgery [4–6]. Tracer uptake is globally and uniformly reduced in patients with TVD and balanced ischemia, leading to a seemingly homogeneous and normal distribution of the tracer in the myocardium, which presents a challenge for visual interpretation. Previous studies of the visual interpretation of SPECT MPI for detecting TVD have reported high false-positive rates in the range of 25% to 35% [7–10]. Currently, summed scores calculated using quantitative perfusion SPECT (QPS) are the most commonly used quantitative parameters in routine SPECT MPI studies. However, reports have also shown that QPS scores perform only modestly in the diagnosis of TVD. For example, in a recent report by Gimelli et al., it was found that the summed stress score and summed difference score provided areas under the curve (AUCs) of 0.79 and 0.69, respectively, using cadmium zinc telluride- (CZT-) based SPECT cameras [11]. Similar studies have reported QPS scores to have variable and unsatisfactory sensitivity values of 46%–75% [12–14]. Such findings seem reasonable given that QPS computes the summed scores based on a comparison between a specific patient study and a population average. If the tracer uptake appears uniform in the myocardium as often seen in TVD patients, QPS scores would tend to be in the normal range, which would make it difficult to discriminate TVD.

In recent years, CZT has been widely adopted as a scintillation material for SPECT cameras. Use of CZT-based cameras is rapidly expanding because of their unique advantages in terms of sensitivity and because they can significantly reduce the tracer dose and scanning time required. Novel CZT-based cameras dedicated for cardiac applications often have a stationary detector design without moving heads [15, 16], consequently allowing new acquisition modes, such as dynamic acquisition. The dynamic capabilities of CZT-based cameras have been tested for absolute quantification of myocardial blood flow and coronary flow reserve. The SPECT-measured coronary flow reserve has been applied to detect TVD in a recent study, showing improved performance (86% sensitivity, 78% specificity) [13]. However, SPECT-based flow quantification is still under investigation and requires more data to verify its clinical performance. Moreover, current protocols for quantitative SPECT rest/stress studies are quite time-consuming because the tracer injection must be performed on table, which offsets the time-saving advantage of CZT-based cameras. As a result, although several reports have shown the accuracy of measurement of coronary flow reserve with SPECT, the nuclear medicine community may still need some time to adopt such methods for routine studies, hence limiting its contribution in the diagnosis of TVD.

In this study, we took the advantage of the stationary detector design of CZT-based cameras by assuming that such a design would allow us to correlate the local signal-to-noise ratio (SNR) with the amount of acquired counts. In tomographic image reconstruction, the relationship between the local SNR and counts on positron emission tomography has been established [17, 18]. In general, the more the counts that are acquired, the higher the local SNR that can be expected from an area of uniform tracer uptake. On the other hand, for SPECT cameras with rotating heads, counts have been dependent on the rotating mechanism and positions of the camera heads. Therefore, it was traditionally assumed that the SNR cannot be directly correlated with the acquired counts for SPECT. Now that the dedicated CZT-cased cameras have a stationary detector arrangement, it is in theory possible to use the local SNR to reflect the counts acquired. Conceptually, the ratio of the stress SNR to the rest SNR should be correlated with the ratio of counts acquired from stress and rest studies. Since the tracer uptake during a stress study is roughly halved in patients with TVD when compared with controls, it may be hypothesized that the SNR during the stress acquisition would be significantly lower in patients with TVD and therefore own the potential to be a discriminative marker. Accordingly, we hypothesized that the stress-to-rest ratio of the SNR (RSNR) would reflect the difference in myocardial blood flow between the rest and stress conditions and be potentially useful for diagnosis of TVD. To investigate this hypothesis, we used a fully automatic segmentation method to delineate the left ventricle (LV) and calculated the RSNR in control subjects and patients with TVD from their Tl-201 scans. Performance of the calculated parameter was evaluated against the reference from coronary angiography (CAG) findings.

## 2. Material and Methods

### 2.1. Acquisition of Clinical Data

This retrospective study was approved by the Institutional Review Board of Chang Gung Memorial Hospital, Linkou, Taiwan. We retrospectively collected clinical data from 102 consecutive subjects. All subjects signed the informed consent for the imaging studies. Due to suspected or preexisting coronary artery disease, these subjects underwent SPECT MPI followed by CAG no later than two months after the initial SPECT scans. The inclusion criterion for the TVD group was at least 50% stenosis in all three major coronary branches or 50% stenosis in the left main stem based on CAG findings [7, 19]. The inclusion criterion for the control group was no more than 50% stenosis in any of the three major coronary branches or in the left main stem. Subjects with previous coronary stenting and those who had previously undergone coronary bypass surgeries were excluded from this cohort. Using these criteria, 61 subjects were allocated to the control group and 41 to the TVD group. The clinical information, including age, gender, New York Heart Association (NYHA) classification, ECG findings, and lab results, was collected for each subject. Left ventricular ejection fraction (LVEF) was measured with echocardiography for all subjects except for one.

SPECT MPI was performed according to the standard stress/rest protocol [20]. Each subject was pharmaceutically stressed with dipyridamole 0.142 mg/kg/min infused slowly over 4 minutes, after which an intravenous injection of 2 mCi of Tl-201 was administered. Five to 10 minutes after injection of the tracer, the subject was scanned using a CZT-based SPECT camera (Discovery NM 530c, GE Healthcare, Little Chalfont, UK) in the supine position. Before starting data acquisition, the technician would use the real-time scintillation images to position the heart as close as possible to the center of the field of view. Once positioning was completed, a gated stress acquisition was started to acquire list-mode data over 5 minutes. The images were reconstructed using three-dimensional iterative Bayesian reconstruction algorithm [21, 22] in a 70 × 70 matrix with 50 slices. The pixel size was set to 4 mm in all three directions. As our SPECT is not equipped with computed tomography, attenuation correction was not performed. Four hours later, the subject was scanned again for acquisition of data at rest. After manually reorienting the images into long-axis and short-axis views, a board-certified nuclear physician used QPS to calculate the summed rest score (SRS), summed stress score (SSS), and summed difference score (SDS) using the built-in software (Myovation for Alcyone, GE Healthcare) [23].

### 2.2. Segmentation of the LV Myocardium

**Figure 1:**
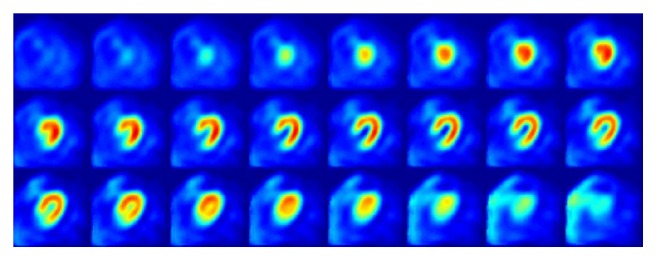
Template images generated from ten male subjects.

We used a segmentation method based on spatial normalization that we have described previously [24]. In brief, this approach spatially normalizes the image volume of an individual to a “template” image set precalculated from cohort data. After the transformation relationship is found, the inverse transformation is applied to the predefined LV mask to find the corresponding mask location over the original image volume for the same individual. In this study, we generated the template using the following procedure. The spatial template was generated from images acquired under resting conditions using the same scanner in 20 subjects separate from the study cohort. Average age of them was 63.8 ± 8.9, 61.8 ± 8.2, and 62.8 ± 8.4 for female, male, and all subjects, respectively. BMI was 25.0 ± 6.0, 30.8 ± 4.0, and 27.9 ± 5.8 for female, male, and all subjects, respectively. These subjects were with negative findings from the SPECT MPI. As none of them underwent further CAG exams, they were not included in the control group of this study. Template images were created separately for men (*n* = 10) and women (*n* = 10). To generate the template for men, one of the ten male subjects was chosen. The images for the other nine subjects were then spatially normalized to this one subject with the software package Statistical Parametric Mapping (SPM, version 8) [25] under MATLAB R2015a (MathWorks Inc., Natick, Massachusetts). The final template for men was generated by averaging all these spatially normalized images, as shown in Figure 1. The template for women was created in the same fashion, using the other ten female subjects. The LV mask was delineated over the template images using the Otsu method [26] separately for male and female subjects.

Segmentation of the LV myocardium for a specific subject was performed as follows. First, the image volume for a specific subject was spatially normalized to the template image for the corresponding sex. The transformation was individually calculated for each subject. Second, this transformation was used to inversely transform the LV mask over the template image into an individual LV mask over the image volume of the specific subject. This inversely transformed LV mask was regarded as the volume of interest (VOI) in the LV myocardium of that subject. All segmentation steps were executed automatically without requiring any manual intervention. The rest and stress images for each subject were segmented to form separate LV VOIs to take into account the fact that the patient's position may be different between the rest and stress acquisitions.

### 2.3. Computation of the RSNR

The RSNR was calculated after the LV myocardial masks were segmented for the rest and stress studies. First, the voxel intensities within the LV VOI of the rest acquisition were taken and used to calculate a mean and standard deviation (SD). SNR_rest_ was then calculated as the mean divided by the SD. SNR_stress_ was calculated in the same way, using the stress acquisition and the segmented LV VOI over the stress acquisition. RSNR was calculating by dividing SNR_stress_ by SNR_rest_. These procedures were performed independently and automatically for all subjects.

### 2.4. Statistical Analysis

As described earlier in this section, the subjects were allocated to the control group or the TVD group groups using the CAG reference criteria for stenosis. The mean (±SD) RSNR was calculated for each group. Student's* t*-test was performed to determine if there was a significant difference in the RSNR between the two groups. Receiver-operating characteristic (ROC) analysis was performed to evaluate the diagnostic performance of RSNR. AUC, accuracy, sensitivity, specificity, and optimal cutoff values were calculated from the ROC curves. A similar statistical analysis was performed for SNR_rest_, SNR_stress_, and the QPS-derived SRS, SSS, and SDS, with mean and SD values calculated for the control group and the TVD groups. ROC analyses were performed for the six parameters (SRS, SSS, SDS, SNR_rest_, SNR_stress_, and RSRN) independent of each other. The parameter (among SRS, SSS, and SDS) with the highest diagnostic accuracy was selected for comparison against the diagnostic performance of RSNR. The sensitivity and specificity of the RSNR and the best-performing score parameter were compared using McNemar's test. A *P* value < 0.05 was considered to indicate a statistically significant difference between these indices. Univariate logistic regression analysis was used to evaluate how these parameters correlate to disease state (without or with TVD). The statistical analysis was performed with SPSS version 21 (IBM, Armonk, NY).

We also evaluated how age, gender, and body mass index (BMI) affect the diagnostic performances of the SNR-based parameters (SNR_rest_, SNR_stress_, and RSRN) and QPS-based parameters (SRS, SSS, and SDS). To evaluate the effect of age, the subjects were divided into two groups: those with ages less than median age and those with ages greater than or equal to the median age. Using the cutoff values determined from the ROC analysis, we examined what the sensitivity, specificity, and accuracy were obtained from those two age groups. The similar analysis was repeated for the male and female subjects, respectively. Lastly, this analysis was performed using BMI as the grouping criterion which separates the subjects into two groups with the median BMI.

## 3. Results

In this retrospective study, a total of 102 subjects were involved. 41 subjects were placed in the TVD group, whereas the other 61 subjects were placed in the control group. Their demographics and cardiac function parameters were summarized in Table 1. Among these parameters, age was the only parameter that showed a significant difference (*P* < 0.05) between the control and TVD groups. As age was not a specific inclusion or exclusion criterion, this difference might simply be due to the relatively small size of study cohort (*n* = 102).

**Figure 2:**
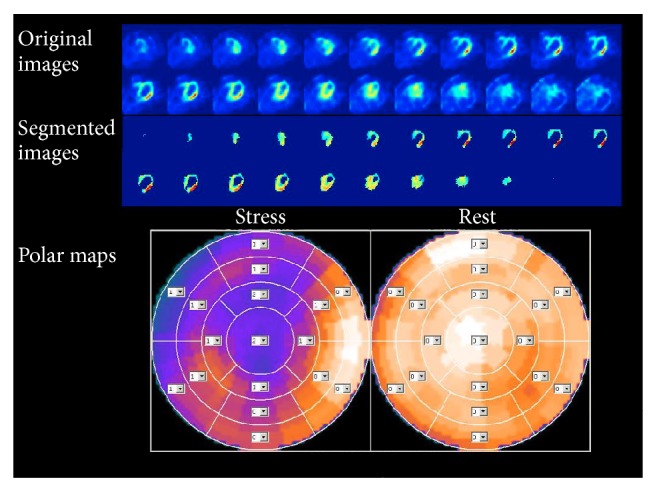
The MPI images of a representative TVD case, a 55-year-old man, which showed obvious ischemic deficits. From top to down, the original axial views of the stress acquisition, the segmented axial views of the stress acquisition, and the polar maps were shown. SSS, SRS, and SDS are 14, 0, and 14, respectively. Although the images show ischemic defects in the LAD and LCX, the segmentation was successful to delineate the LV. The SDS was 14 and the RSNR was 0.71. CAG found stenosis of 86% in the LAD, 82% in the LCX, and 68% in the RCA. CAG, coronary angiography; LAD, left anterior descending; LCX, left circumflex; RCA, right coronary artery; RSNR, stress-to-rest ratio of the signal-to-noise ratio; SDS, summed difference score.

**Figure 3:**
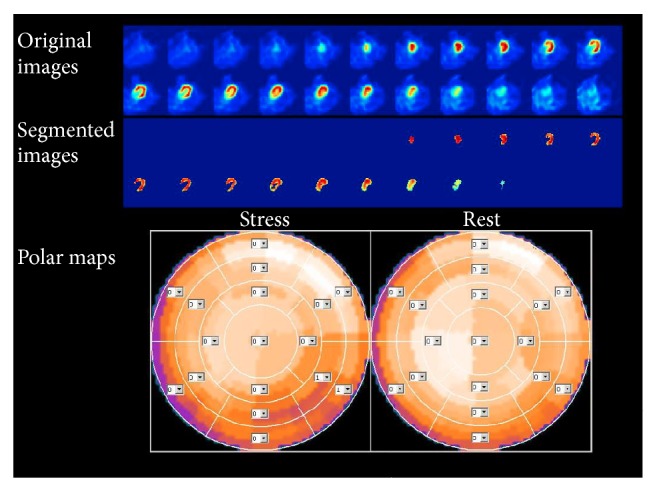
The stress MPI images of a representative TVD case, a 64-year-old woman, which showed uniform tracer uptake in the myocardium. From top to down, the original axial views of the stress acquisition, the segmented axial views of the stress acquisition, and the polar maps were shown. SSS, SRS, and SDS are 2, 0, and 2, respectively. The RSNR was 0.91, indicating a low tracer uptake in the stress study and high possibility of present TVD. The CAG identified stenosis of 61% in the LAD, 69% in the LCX, and 62% in the RCA.

Using the segmentation method described above, LV VOIs were delineated from the rest and stress acquisitions for all subjects. We confirmed visually that the automatic segmentation appropriately delineated the LV myocardium in all subjects, including those with obvious ischemic defects or dilated myocardium. Figures 2 and 3 showed the segmentation results of two representative cases. The QPS-derived SRS, SSS, and SDS and the proposed SNR_rest_, SNR_stress_, and RSNR were calculated from rest/stress Tl-201 images for all subjects in both study groups. The statistics and ROC results of the QPS-derived parameters were summarized in Table 2. The statistics and ROC results of SNR-based parameters were summarized in Table 3. The SSS, SDS, SNR_stress_, and RSNR values were found to be significantly different between the two study groups (*P* < 10^−5^). Those four parameters also showed a significant predicting power for TVD subjects (*P* < 10^−3^) in the simple logistic regression analysis results shown in Table 4. RSNR was lower in patients with TVD (0.83 ± 0.15) than in control subjects (1.06 ± 0.17) as expected because of the reduced uptake during the stress acquisition for patients with TVD. This reduction seemed to be mainly due to the reduction of SNR_stress_ since SNR_rest_ did not show a significant difference between the control and TVD groups.

**Figure 4:**
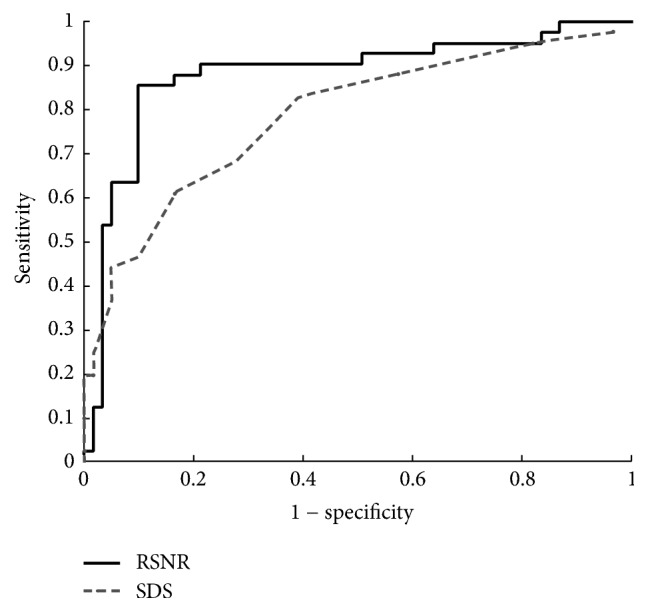
ROC analysis. The ROC curves for the proposed parameter RSNR and SDS are shown on the same plot. RSNR provides a better diagnostic performance than the SDS, with AUC of 0.88 versus 0.75. AUC, area under the curve; ROC, receiver-operating characteristic; RSNR, stress-to-rest ratio of the signal-to-noise ratio; SDS, summed difference score.

After we evaluated the statistics of computed parameters, we applied the QPS-based and SNR-based parameters to discriminate the TVD patients from control subjects. In the results of the ROC analysis shown in Tables 2 and 3, RSNR performed the best in discriminating subjects with and without TVD, with a sensitivity of 85%, a specificity of 90%, an accuracy of 88%, and an AUC of 0.88. The cutoff value for RSNR was found to be 0.94. For QPS scores, the accuracy of the SRS, SSS, and SDS was 59%, 70%, and 71%, respectively. The SDS was found to be the best-performing QPS parameter. McNemar's test showed that, compared to SDS, RSNR had significantly better sensitivity (85% versus 68%, *P* < 0.05) and significantly better specificity (90% versus 72%, *P* < 0.05). The ROC curves for RSNR and SDS are plotted in Figure 4.

We applied the cutoff determined from the ROC analysis to the age-, gender-, and BMI-specific discrimination of the TVD subjects for the QPS-based and SNR-based parameters. The performances in terms of the sensitivity, specificity, and accuracy were summarized in Table 5. For RSNR, age, gender, and BMI did not make much differences in the discrimination performances, in general. Discrimination accuracy with RSNR remained between 86% and 90%. However, we did find that diagnostic sensitivity was lower (75%) in the high-BMI group than the low BMI group (95%).

## 4. Discussion

In this study, we aimed to address the challenge of detecting multivessel disease using SPECT MPI. The literature indicates that visual interpretation on SPECT may lead to a high false-negative rate and therefore has unsatisfactory sensitivity in detecting TVD. Quantification of absolute flow and flow reserve has been shown to be useful in assisting the diagnosis of TVD with positron emission tomography [27, 28] and magnetic resonance imaging [3, 29]. Although SPECT-based quantification of flow has also been investigated in recent years [13, 19, 30–34], its adoption for routine studies is still limited due to the prolonged acquisition time and other technical difficulties. In the present study, we tested our hypothesis that TVD may be detected with a simple parameter, that is, the ratio between local SNRs in the LV myocardium from the rest and stress studies. We used Tl-201 images acquired from 102 subjects to evaluate this hypothesis. Our current data support our working hypothesis concerning the usefulness of RSNR. Using our proposed data processing scheme, the RSNR can be calculated automatically with 85% sensitivity, 90% specificity, and 88% accuracy when used to detect TVD. By way of comparison, the SDS was found to be the most accurate parameter among the three summed scores in our data, but even SDS could only discriminate patients with TVD from controls with 68% sensitivity, 72% specificity, and 71% accuracy, which is similar to the performance previously reported for QPS-derived scores [11–14]. Comparing the diagnostic performance of the two parameters, our data show that the proposed RSNR, calculated with a fully automatic procedure, has the potential to advance the sensitivity and accuracy in the diagnosis of TVD.

We have also evaluated whether age, gender, and BMI would affect the diagnostic performances of RSNR. We found that age does not seem to affect the diagnostic performances with the sensitivity/specificity/accuracy being 81/91/88 (%) in the lower age group and 88/88/88 (%) in the higher age group. Similarly, the same pattern was observed in the two gender groups. The BMI groups, on the other hand, did show a slightly different pattern than age or gender. We found that, with the median BMI of 25.3 as the cutoff to separate the subjects, the sensitivity/specificity/accuracy was found to be 95/87/90 (%) for the lower BMI group and 75/94/86 (%) for the higher BMI group. Although the accuracy and specificity were not much different than those from the whole study population, there was an obvious drop of sensitivity to 75% in the higher BMI group. We presume that this drop might be due to the lack of attenuation correction in our study. Since the attenuation is dependent on the patient size, BMI may have a nonnegligible effect over the RSNR values. One potential remedy for this is to establish different RSNR cutoff values for low- and high-BMI groups separately, if a larger cohort may be obtained in the future. Alternatively, if computed tomography is available on the SPECT scanner, it would be useful to evaluate how attenuation correction affects the RSNR values and how RSNR performs in diagnosing TVD when attenuation correction is involved in the image reconstruction.

Although our data seem promising, the proposed method still requires further evaluation before it can become a routine diagnostic tool. The study cohort (*n* = 102) was only of a modest size in this work. Data from a larger cohort will be useful to confirm the diagnostic performance of this method. Further, a multicenter trial will be helpful in determining the extent to which the RSNR is dependent on the scanners, protocols, and reconstruction parameters used. The RSNR cutoff for different tracers may also differ and requires further validation. Myocardial segmentation may be conducted differently using other automatic LV segmentation techniques [35–37], so the effects of different segmentation methods also need to be investigated. In the present study, the RSNR cutoff was the same for both sexes in our current study. How RSNR values vary according to race, ethnicity, sex, and age requires more comprehensive evaluation in the future. Lastly, because there is no computed tomography in our SPECT camera, attenuation correction was not performed. Scatter correction was not performed either, as it was not provided by the scanner vendor. A previous report on the CZT camera performances has shown that the CZT SPECT has a roughly twofold increase in energy resolution that is capable of a better scatter rejection than conventional SPECT cameras [22]. The exact effects of attenuation and scatter correction on the RSNR remain to be studied in the future.

## 5. Conclusion

A novel parameter based on the stress-to-rest ratio of the SNR, namely, RSNR, has been proposed and shown to be a potentially useful parameter for detecting TVD with CZT-based SPECT MPI. According to our data, this fully automatic method provides a high sensitivity of 85% and high specificity of 90% in discriminating TVD. Patient size does seem to cause a drop in the detection sensitivity with RSNR to 75% in the high-BMI group, but the discrimination accuracy is generally independent of the patient size, age, or gender. Although further studies are required to evaluate the clinical value of RSNR, it points to a robust and quantitative way to assist the diagnosis of TVD with SPECT MPI.

## Figures and Tables

**Table 1:** Demographic characteristics of study participants (*n* = 102).

Characteristic	All	Control	TVD
*n*	102	61	41
Age (yr)	62.6 ± 12.5	60.3 ± 13.3	66.0 ± 10.5^*∗*^
Female sex	37	19	18
Hypertension	78	39	39
Body mass index (kg/m^2^)	25.9 ± 4.9	26.5 ± 5.2	25.1 ± 4.4
Dyslipidemia	48	24	24
Diabetes	39	15	24
Tobacco use	25	18	7
Angina	74	44	30
LVEF (%)	57.4 ± 17.2	55.7 ± 18.2	59.9 ± 15.4
Arrhythmia	22	16	6
ECG ST change	33	17	16
NYHA class			
I	76	41	35
II	17	14	3
III	6	5	1
IV	3	1	2

TVD, triple-vessel disease; LVEF, left ventricular ejection fraction; ECG, electrocardiography; NYHA, New York Heart Association; *∗* denotes a statistical significance in the mean difference between the control and TVD subjects (*P* < 0.05).

**Table 2:** Statistics and the ROC results of the QPS scores.

Parameter	SRS			SSS			SDS		
	All	Control	TVD	All	Control	TVD	All	Control	TVD
Mean	2.12	2.07	2.2	5.44	3.52	8.29	3.28	1.46	6.00
SD	2.67	2.6	2.81	5.24	3.78	5.83	4.62	2.38	5.72
Sensitivity		34			78			68	
Specificity		75			64			72	
Accuracy		59			70			71	
AUC		0.49			0.77			0.75	
Cutoff		2.5			3.5			2.5	
*P* value	0.81			*P* < 10^−5^			*P* < 10^−5^		

*P* value is derived from the *t*-test of the control versus TVD subjects. ROC, receiver-operating characteristic; QPS, quantitative perfusion single-photon emission computed tomography; SRS, summed rest scores; SSS, summed stress scores; SDS, summed difference scores; AUC, area under the curve.

**Table 3:** Statistics and the ROC results of the image-derived SNR at rest and stress and the ratio of the SNR_stress_, SNR_rest_, and RSNR.

Parameter	SNR_rest_			SNR_stress_			RSNR		
	All	Control	TVD	All	Control	TVD	All	Control	TVD
Mean	4.09	4.03	4.18	3.92	4.24	3.44	0.97	1.06	0.83
SD	0.70	0.73	0.66	0.85	0.83	0.65	0.2	0.17	0.15
Sensitivity		83			76			85	
Specificity		43			75			90	
Accuracy		59			75			88	
AUC		0.57			0.78			0.88	
Cutoff		3.74			3.7			0.94	
*P* value	0.26			*P* < 10^−5^			*P* < 10^−5^		

*P* value is derived from the *t*-test of the control versus TVD subjects. SNR, signal-to-noise ratio; SNR_rest_, signal-to-noise ratio at the rest study; SNR_stress_, signal-to-noise ratio at the stress study; RSNR, stress-to-rest ratio of the signal-to-noise ratio.

**Table 4:** Univariate logistic regression results of selected parameters.

Variable	Odds ratio	*P* value	95% CI
Age	1.04	0.03	1.00–1.08
Sex	1.73	0.19	0.76–3.93
BMI	0.94	0.19	0.87–1.03
SRS	1.02	0.81	0.88–1.18
SSS	1.24	<0.001	1.12–1.38
SDS	1.40	<0.001	1.19–1.65
SNR_rest_	1.39	0.26	0.78–2.46
SNR_stress_^§^	4.48	<0.001	2.21–9.11
RSNR^§^	33432.17	<0.001	469.91–2378545.76

^§^For  SNR_stress_ and RSNR, since their mean is lower in the TVD group, the logistic regression was performed using (SNR_stress_) and (RSNR) against the TVD status.

**Table 5:** Diagnostic performances of QPS scores and SNR parameters in specific age, gender, and BMI groups.

Variable	*n*	SRS	SSS	SDS	SNR_rest_	SNR_stress_	RSNR
Age							
<62.5	51	25/74/59	69/63/65	75/74/75	81/43/55	69/83/78	81/91/88
≥62.5	51	40/71/59	84/65/75	64/69/67	84/42/63	80/66/73	88/88/88
Sex							
Male	65	35/76/62	83/64/71	65/71/69	87/29/49	74/86/82	87/90/89
Female	37	33/74/54	72/63/68	72/74/73	78/74/76	78/53/65	83/89/86
BMI							
<25.3	51	29/70/53	81/63/71	62/73/69	86/43/61	71/83/78	95/87/90
≥25.3	51	40/81/65	75/65/69	75/71/73	80/42/57	80/68/73	75/94/86

Numbers are presented as percentages of sensitivity/specificity/accuracy; for age and BMI, the cutoff for grouping was determined from their medians.
